# Analysis of the middle region of the pharynx in adolescents with different anteroposterior craniofacial skeletal patterns

**DOI:** 10.1590/2177-6709.24.5.060-068.oar

**Published:** 2019

**Authors:** Priscilla de Almeida Solon de Mello, Bruna Caroline Tomé Barreto, Ligia Vieira Claudino, Claudia Trindade Mattos, Guido Artemio Marañón-Vásquez, Mônica Tirre de Souza Araújo, Eduardo Franzotti Sant’Anna

**Affiliations:** 1 Universidade Federal do Rio de Janeiro, Departamento de Odontopediatria e Ortodontia (Rio de Janeiro/RJ,Brazil).; 2 Universidade Federal Fluminense, Departamento de Ortodontia (Niterói/RJ,Brazil).

**Keywords:** Pharynx, Cone-beam computed tomography, Sleep apnea syndromes, Diagnosis.

## Abstract

**Objective:**

To assess the volume and morphology of the middle region of the pharynx (MRP) in adolescents with different anteroposterior craniofacial skeletal patterns.

**Methods:**

One hundred twenty-six patients (56 male and 70 female), who had cone-beam computed tomography (CBCT) within their records, were selected for this cross-sectional study. Participants were classified, according to their ANB angle value, in Class I (1^o^ ≤ ANB ≤ 3^o^), Class II (ANB > 3^o^) and Class III (ANB < 1^o^). The total volume (tV), minimum axial area (AxMin) and morphology of the MRP and its subdivisions - velopharynx (VP) and oropharynx (OP) - were characterized by CBCT and 3-dimensional image reconstruction software. Intergroup comparisons were performed by ANOVA and Tukey *post-hoc* tests. Correlations between tV and Axmin with the ANB angle values were tested using linear regression analysis, considering sex as covariable.

**Results:**

Statistically significant difference between groups were observed in tV only for the VP region; Class II individuals presented significantly lower tV (6863.75 ± 2627.20 mm^3^) than Class III subjects (9011.62 ± 3442.56 mm^3^) (*p*< 0.05). No significant differences were observed between groups for any other variable assessed, neither in MRP nor in the OP region (*p*> 0.05). A significant negative correlation was evidenced between tV and Axmin and the ANB angle values; sexual dimorphism was observed for some variables.

**Conclusions:**

Class II subjects have smaller tV in the VP region. tV and Axmin tend to decrease in all evaluated regions when the ANB angle values increase.

## RESUMO

**Objetivo:**

avaliar o volume e a morfologia da porção média da faringe (PMF) em adolescentes com diferentes padrões esqueléticos no sentido anteroposterior.

**Método:**

para esse estudo transversal, foram selecionados cento e vinte e seis pacientes (56 homens e 70 mulheres) que tinham tomografia computadorizada de feixe cônico (TCFC) em seus registros. Os participantes foram classificados de acordo com o valor do ângulo ANB, em Classe I (1^o^ ≥ ANB ≤ 3^o^), Classe II (ANB > 3^o^) e Classe III (ANB < 1^o^). O volume total (Vt), a área axial mínima (AxMin) e a morfologia da PMF e de suas subdivisões - velofaringe (VF) e orofaringe (OF) - foram avaliados na TCFC e em *software* de reconstrução tridimensional (3D) de imagens. As comparações intergrupos foram realizadas por meio dos testes ANOVA e *post-hoc* de Tukey. As correlações entre os valores de Vt e Axmin das vias aéreas com os valores do ângulo ANB foram testadas por meio de análise de regressão linear, considerando-se o sexo como uma covariável.

**Resultados:**

diferença estatisticamente significativa entre os grupos foi observada apenas no Vt da VF; indivíduos Classe II apresentaram Vt significativamente menor (6.863,75 ± 2.627,20 mm^3^) do que indivíduos Classe III (9.011,62 ± 3.442,56 mm^3^) (*p*< 0,05). Não foram observadas diferenças significativas entre os grupos em nenhuma outra variável avaliada, nem na PMF, nem na OF (*p*> 0,05). Foi evidenciada uma correlação negativa significativa entre Vt e Axmin e os valores do ângulo ANB; dimorfismo sexual foi observado para algumas variáveis.

**Conclusões:**

indivíduos Classe II apresentam o menor Vt da VF. O Vt e a Axmin tenderam a diminuir quando os valores do ângulo ANB aumentaram, em todas as regiões avaliadas.

## INTRODUCTION

For more than a century, diverse aspects of the interrelation between respiratory function and craniofacial morphology have been studied in Orthodontics field. Several authors stated that there is a cause-effect relationship between respiratory disorders and unbalanced growth and development of the craniofacial complex.^1^ ^-^ ^5^ However, the literature is still controversial in how the morphology of the upper airways (UA) and variations in the airflow would influence facial features.^6^ ^-^ ^14^ Methodological limitations, multifactorial etiology of malocclusion, influence of the respiratory phase and tongue position on the UA dimensions, limitations of the evaluation method, absence of consensus in the literature for the determination of the limits and regions of interest evaluated, lack of longitudinal studies assessing the UA,^8^ ^,^ ^15^, among other factors, make the information not conclusive in this topic.

For some years, studies used lateral cephalometric radiographs to assess the UA. Although they provide relevant information, radiographs reproduce 3-dimensional structures in a 2-dimensional way, so detailed anatomy of the surrounding soft tissue, transversal sections areas and UA volume cannot be evaluated.^16^ ^,^ ^17^ The introduction of cone-beam computed tomography (CBCT) in Dentistry at the end of the 90s allowed to reproduce high-quality 3D images and, consequently, more accurate characterization of UA.^15^ ^,^ ^18^ CBCT has been indicated as a reliable and reproducible method to perform these evaluations.^18^ ^,^ ^19^ From a radiation protection point of view, CBCT reduces significantly the radiation dose when compared with traditional medical computed tomography, becoming equivalent to a full-mouth series using periapical radiographs.^20^ Consequently, this technology has become widely accepted for the 3D analysis of the UA, due to advantages related to, among others, high accuracy, relatively low costs and short acquisition time.^14^ ^,^ ^15^

Considering that functional improvement of the craniofacial complex is an objective of the orthodontic treatment, UA characterization and monitoring are fundamental considerations that clinicians should think about in order to guarantee normal development in growing patients, identify the risk of presenting respiratory disturbances and avoid potential collapse of the pharyngeal airspace during treatment. A preliminary study, conducted by the present research group,^13^ characterized the volume and morphology of the pharyngeal airway in adolescents, demonstrating that Class II subjects presented significant differences with the other skeletal patterns. Thus, the present study aimed to replicate this preliminary study in a larger sample, characterizing, by CBCT and 3D image reconstruction software, the total volume (tV), minimum axial area (AxMin) and morphology of the middle region of the pharynx (MRP) and its subdivisions, in adolescents according to their anteroposterior craniofacial skeletal pattern.

## MATERIAL AND METHODS

Research Ethics Committee of the Institute for Studies in Collective Health of the Federal University of Rio de Janeiro approved the protocol of this study (nº 110/2011), which was performed according to the Declaration of Helsinki and its amendments, and respecting ethical legal principles regulated by local resolution (CNS 196/96). Methods were based on a previously published preliminary study.^13^

A sample size calculation was performed based on the highest standard deviation from a previous study,^10^ and the formula described by Pandis.^21^ A sample of at least 39 patients would be necessary in each group (Class I, Class II and Class III) to detect a difference of 50 mm^2^ in the AxMin of the MRP, considering a test power of 90% (β = 0.1) and significance level of 5% (α = 0.05). The selection of a difference of 50 mm² was based on the assumption that individuals with AxMin lower than 52 mm² are more likely to develop Obstructive Sleep Apnoea/Hypopnea Syndrome (OSAHS), and with AxMin between 52 mm^2^ and 110 mm^2^, present moderate probability of presenting this condition.^13^ ^,^ ^22^

Dental records of patients from the Orthodontics graduate clinic at the School of Dentistry from Federal University of Rio de Janeiro were assessed for eligibility. Inclusion criteria consisted in: (1) patients aged between 13 and 20 years old; (2) having pretreatment CBCT (DICOM files) previously requested for diagnostic and planning purposes; (3) no previous orthodontic treatment or any other therapy that could interfere with the normal maxillomandibular growth and development, before CBCT acquisition; (4) no systemic and/or oral diseases; (5) no airway pathology; (6) no craniofacial congenital or syndromic anomalies; (7) craniocervical angle between 90^o^ and 110^o^ during acquisition of CBCT records; (8) no severe mandibular hyperdivergence or hypodivergence (19^o^ ≤ FMA ≤ 30^o^). One hundred twenty-six patients (56 male and 70 female) fulfilled the inclusion criteria and were selected for this cross-sectional study. Participants and/or parents or caretakers were invited to participate and informed consent was obtained before analyses.

All CBCT scans selected for the present study had been acquired following a standardized protocol for image acquisition (90 kV, 10 mA, FOV of 18.4 x 20.6 cm, voxel size of 0.3 mm and 24’’ of scanning) and positioning of the patients (Frankfort horizontal plane parallel to the ground, in maximum intercuspation and without swallowing during the acquisition),^13^ using the same tomographic equipment Kodak^®^ 9500 Cone Beam 2D System (Carestream Health, Rochester, NY, EUA). Three-dimensional images were assessed on Dolphin Imaging^®^ software, version 11.8 Premium (Dolphin Imaging, Chatsworth, CA, USA) and specific tools were used for standardization purposes. 

Head position was oriented on virtual space according to axial plane (going through the right and left orbital reference points and right porion), coronal plane (perpendicular to the axial plane passing through both orbital points) and sagittal plane (perpendicular to the previous planes). These planes served as fixed bases to perform measurements. All analyses were realized by experienced evaluators, previously trained and calibrated.

FMA and craniocervical angles were measured to confirm inclusion criteria on two-dimensional lateral cephalometric radiographs created from CBCT scans. Similarly, ANB angle was determined and patients were classified, according to its values, into three groups: (1) Class I (1^o^ ≤ ANB ≤ 3^o^), (2) Class II (ANB > 3^o^) and (3) Class III (ANB < 1^o^). 

**Figure 1:**
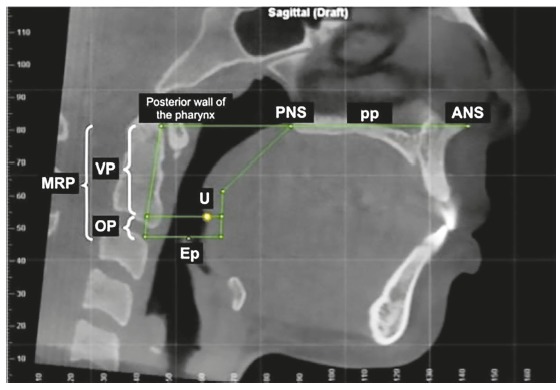
Definition of assessed regions: pp = palatal plane, ANS = anterior nasal spine, PNS = posterior nasal spine, U = the lowest point of the uvula, Ep = the most superior point of the epiglottis, MRP = middle region of the pharynx, VP = velopharynx, OP = oropharynx.

UA assessment was performed using the tool for airway volume calculation in the 3D mode of the software. Patient’s head was reoriented with the palatal plane (pp) parallel to the ground, and the pharyngeal airspace area of interest was defined in the sagittal slice. Technical and anatomical limits of each region assessed in this study were determined as follows (Fig 1): (a) MRP: upper limit - pp extended until the posterior wall of the pharynx, lower limit - plane parallel to pp that intersects the most superior point of the epiglottis (Ep); (b) velopharynx (VP): upper limit - pp extended until the posterior wall of the pharynx, lower limit - plane parallel to pp that intersects the lowest point of the uvula (U); (c) oropharynx (OP): upper limit - plane parallel to the pp that intersects point U; lower limit - plane parallel to pp that intersects point Ep.

**Figure 2:**
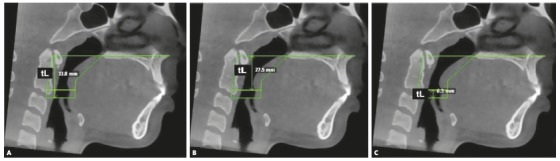
tL measurement. A) tL of the MRP; B) tL of the VP; C) tL of the OP.

**Figure 3:**
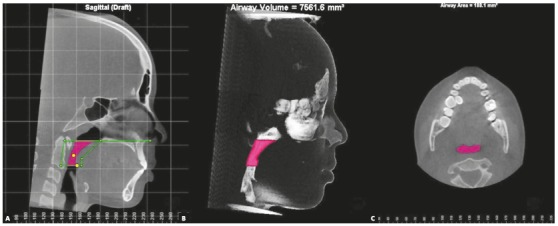
Evaluation of tV and AxMin: A) definition of pharyngeal airspace of interest (i.e. VP); B) tV visualized in the sagittal plane; C) AxMin visualized in the axial plane.

The total length (tL) (mm) of each region was defined as the distance between their upper and lower limits (line perpendicular to both planes), using a specific tool that allows measurement of linear distances (Fig 2). The tV (mm^3^) and AxMin (mm^2^) were automatically determined by specific commands on the software (Fig 3). The mean area of each segment was then calculated using the following ratio: mean area = tV/tL. Similarly, morphological characterization of each region assessed was determined by calculation of the following ratio: AxMin/mean area.^13^ ^,^ ^23^ ^,^ ^24^ This ratio allows demonstrating whether the area distribution of each UA segment was uniform or irregular. UA morphology was considered more irregular if the value obtained from the ratio was lower.

### Statistical analysis

The Kolmogorov-Smirnov test was applied to assess the normality of the data. Differences between groups were verified by ANOVA and Tukey *post-hoc* tests. Correlations between values for airway volumes and AxMin, and ANB angle values were tested using linear regression analysis, considering sex as co-variable. All analyses were performed in the software SPSS 17.0 (SPSS Inc., Chicago, Illinois, USA) with a significance level of 5% (α = 0.05). 

### Method error

Repeated measures (two-week interval) of 21 patients randomly selected were correlated to determine both intra and inter-examiner agreement using the intraClass correlation coefficient (ICC). The method was considered reliable when ICC was greater than or equal to 0.9 for each measurement.

## RESULTS

ICC demonstrated intra and inter-examiner agreements higher than 0.9 for all measures assessed on the present study.

One hundred twenty-six pretreatment CBCT images were analyzed. Patients were divided in the following groups: Class I (41 patients, 17 male and 24 female), Class II (45 patients, 26 male and 19 female) and Class III (40 patients, 13 male and 27 female). Data regarding age and cephalometric values (ANB, FMA, and craniocervical angles) according to craniofacial skeletal classification are presented in Table 1.

**Table 1:** Means (SD) of age and cephalometric angle values according to the skeletal Class.

	Class I (n=41)	Class II (n=45)	Class III (n=40)
	Mean (SD)	Mean (SD)	Mean (SD)
Age (years)	14.54 (1.94)^a^	14.62 (1.91)^a^	15.73 (2.09)^b^
ANB (degrees)	2.14 (0.63)^a^	6.14 (2.39)^b^	-2.01 (2.39)^c^
FMA (degrees)	24.40 (3.29)^a^	24.76 (3.17)^a^	23.76 (3.36)^a^
Craniocervical (degrees)	98.87 (5.88)^a^	101.96 (5.37)^b^	98.43 (6.38)^a^

^a,b,c^ Different letters, on the same line, indicate statistically significant difference (p < 0.05).

Inter-group comparisons of measures on dimensional characterization and morphology of UA on MRP, VP, and OP are shown in Tables 2, 3 and 4, respectively. Statistically significant differences between groups were observed in tL and tV only for VP region (*p*< 0.05). Class II individuals presented significantly lower tL and tV, when compared with Class III subjects (Table 3). No significant difference was observed between groups for any other measure evaluated, neither in MRP nor in OP (*p*> 0.05) (Tables 2 and 4, respectively). Morphologically, total MRP showed a less uniform distribution of the airway for all groups, when compared with VP and OP regions. There were no significant differences between UA morphology of groups for any region (*p*> 0.05).

**Table 2:** Inter-group comparisons of UA measures for MRP

MRP	Class I (n=41)	Class II (n=45)	Class III (n=40)
	Mean (SD)	Mean (SD)	Mean (SD)
Total length (mm)	45.88 (5.93)^a^	44.56 (5.50)^a^	45.95 (6.46)^a^
Total volume (mm^3^)	11776.01 (5239.32)^a^	10838.97 (3754.19)^a^	13122.88 (5287.84)^a^
AxMin (mm^2^)	161.57 (81.58)^a^	170.41 (73.12)^a^	194.39 (88.33)^a^
Morphology (AxMin/mean area)	0.62 (0.14)^a^	0.68 (0.12)^a^	0.68 (0.13)^a^

^a^ Different letters, on the same line, indicate statistically significant difference (p < 0.05).

**Table 3:** Inter-group comparisons of UA measures for VP region

VP	Class I (n=41)	Class II (n=45)	Class III (n=40)
	Mean (SD)	Mean (SD)	Mean (SD)
Total length (mm)	29.21 (4.19)^a,b^	28.08 (3.87)^a^	30.21 (3.41)^b^
Total volume (mm^3^)	8138.91 (3371.74)^a,b^	6863.75 (2627.20)^a^	9011.62 (3442.56)^b^
AxMin (mm^2^)	199.80 (100.10)^a^	183.99 (77.58)^a^	220.59 (105.48)^a^
Morphology (AxMin/mean area)	0.70 (0.15)^a^	0.86 (0.94)^a^	0.72 (0.12)^a^

^a,b^ Different letters, on the same line, indicate statistically significant difference (p < 0.05).

**Table 4:** Inter-group comparisons of UA measures for OP region

OP	Class I (n=41)	Class II (n=45)	Class III (n=40)
	Mean (SD)	Mean (SD)	Mean (SD)
Total length (mm)	16.67 (5.41)^a^	16.49 (5.68)^a^	15.74 (6.59)^a^
Total volume (mm^3^)	3658.56 (2178.54)^a^	3784.67 (1794.79)^a^	4012.18 (2460.78)^a^
AxMin (mm^2^)	182.33 (89.25)^a^	193.16 (76.76)^a^	216.77 (97.37)^a^
Morphology (AxMin/mean area)	0.83 (0.10)^a^	0.82 (0.10)^a^	0.86 (0.10)^a^

^a^ Different letters, on the same line, indicate statistically significant difference (p < 0.05).

**Figure 4:**
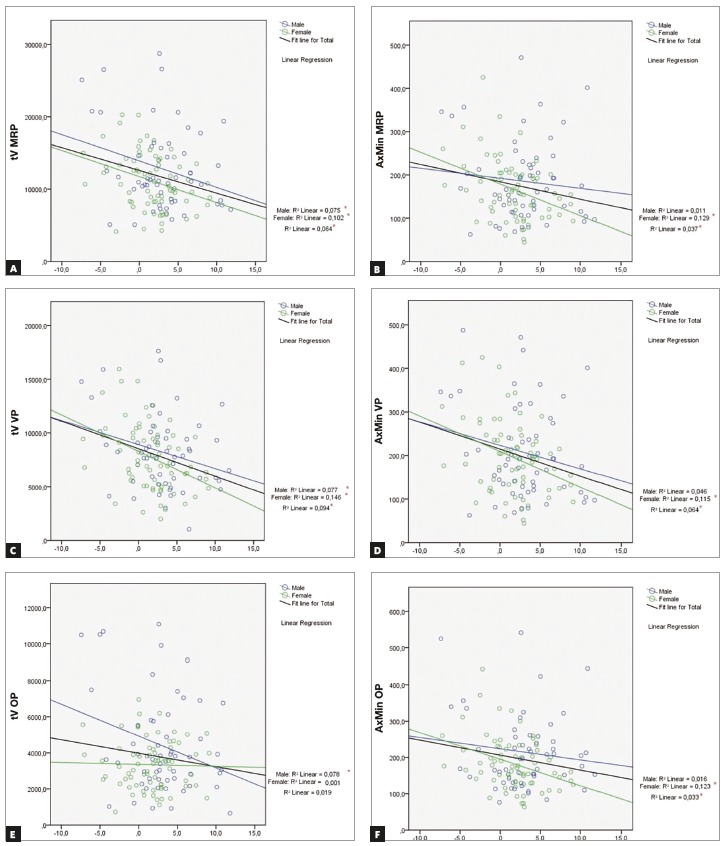
Plots showing linear regression analysis. A and B) correlation between tV and AxMin, and ANB angle values for MRP; C and D) correlation between tV and AxMin, and ANB angle values for VP; E and F) correlation between tV and AxMin, and ANB angle values for OP. * Indicates statistically significant correlation (p < 0.05).

Linear regression analysis evidenced a negative correlation between the variables assessed. tV and AxMin tended to decrease in all evaluated regions when ANB angle values increased (Fig 4). For the variable tV, correlations were significant for the entire sample and both subgroups (male and female) in MRP and VP region (Fig 4A and 4C, respectively). In the OP region, the correlation was significant only for male sex (Fig 4E). For the variable AxMin, the analysis demonstrated a significant correlation for the entire sample and female subgroup on the three evaluated regions (Figs 4B, 4D, and 4F).

## DISCUSSION

Although during the last decades, several studies demonstrated the existing relationship between UA dimensions and different sagittal craniofacial skeletal patterns,^6,9,10,12,13,24,25^ some relevant aspects of this subject still need to be elucidated.^8^ Literature remains controversial in relation to what UA regions of interest are really susceptible to be affected by the craniofacial morphology (or vice versa). 

Regions analyzed in the present study were chosen based on previous results of two preliminary studies assessing UA as a whole.^6^ ^,^ ^13^ Although one of them found a significant difference between groups with different ANB angles for the tV only in the total dimension UA,^6^ the other one, conversely, observed that significant changes in the subdivisions of pharyngeal airspace occurred, VP and OP regions.^13^ Based on this, the present study aimed to analyze, by CBCT images, the MRP and its subdivisions, VP and OP, on adolescents, divided into Class I, Class II and Class III groups, according to their ANB angle values. 

Methods were replicated according to a previous study,^13^ assessing a larger homogeneous sample. Only normodivergent individuals were included for analysis, based on a previous research that compared 197 CBCT images to evaluate UA in Class II individuals with different vertical skeletal patterns and Class I individuals, and did not observe significant differences between groups.^26^ Besides that, excluding extremely hypodivergent or hyperdivergent patients would allow us to have a more homogeneous sample.

Regarding the limits of the segments assessed, there is no consensus in the literature for this topic, thus, measures standardization is challenging. Similarly to previous studies, we used point U and Ep as guides for segmentation,^6^ ^,^ ^27^ because resulting subdivisions are more likely to suffer morphological changes according to skeletal Class. On the other hand, even though using the ANB angle as classification method is questionable, it has been stated as the most reliable indicator to predict maxillomandibular relationship and the main anteroposterior cephalometric measure, expressing a significant relationship with UA volume.^6,27^ Therefore, participants in the present study were classified according to their ANB angle value.

MRP showed no significant differences between groups for any measure. These results were different from those reported by Alves et al,^12^ where Class II patients (ANB >5^o^) presented significantly lower values for tV and AxMin, when compared with Class I individuals (2^o^ ≤ ANB ≤ 5^o^).^12^ This could be due to differences in the parameters to classify skeletal Class and heterogeneity regarding FMA values of the sample. El and Palomo^10^ obtained similar results on tV for Class II, compared to Class I and Class III patients. Although the classification according to ANB values was the same as the one chosen for the present study, the definition and nomenclature of limits and regions of the UA used were different, making it difficult to properly perform comparisons. Similarly, Kim et al,^6^ using a different definition for regions assessed, demonstrated that retrognathic patients have a tV significantly smaller than that of patients with a normal anteroposterior skeletal relationship. It is important to mention that although our results were not significant, Class II patients also presented lower measures for tL and tV.

Regarding subdivisions, significant differences were observed only on VP for tL and tV between Class II and Class III patients. Similarly, Jayaratne and Zwahlen,^24^ using the same parameters and limits to determine VP and OP (retropalatal and retroglossal regions, respectively), demonstrated significant differences between Class II and Class III patients regarding tV and AxMin; however, for tL and morphology, differences were not significant. Parameters of ANB angle values to classify the groups were not clearly described, which could explain disagreement with some results of the present study. Kim et al^6^ did not verify a significant difference between groups assessed (only Class I and II) regarding UA subregions volumes. When compared with the reference preliminary study by Claudino et al,^13^ our results did not confirm all previous findings. For VP, Class II individuals presented a significantly lower AxMin and an increased morphological variation, compared to Class I and Class III; and for OP, AxMin for Class II individuals were significantly lower than for Class III patients.^13^

Linear regression analysis evidenced that tV and AxMin tended to decrease in all evaluated regions when ANB angle values increase, with slight sexual dimorphism for some variables. These results confirmed previous findings of the reference preliminary study.^13^ Considering that tV and AxMin are important parameters to determine predisposition to or presence of OSAHS,^22^ an increased ANB may be considered a risk factor for this condition. It is important to mention that the patients evaluated were aged between 13 and 20 years old, a period of stable growth of UA structures.^13^ Aging is characterized by augmentation of tissues surrounding UA, with consequent narrowing.^28^ Besides that, risk factors for OSAHS usually increase in adult life, such as obesity, predisposing older individuals with specific skeletal patterns to develop obstructive sleep apnea.^29^

Orthodontists must take this information into consideration not only for diagnostic purposes, but also for treatment planning. It has been proven that orthodontic treatment and consequently changes on the patients’ occlusion and surrounding soft tissues can modify UA dimensions. Chen et al^30^ demonstrated that extensive incisors retraction on biprotrusion cases can promote reduction on the space for the tongue in the sagittal direction, which would press it against the soft palate, resulting in UA adaptation and, consequently, narrowing of them.

Our results have high clinical relevance. This knowledge will allow the orthodontist to choose the best treatment option for each patient, avoiding planning that could compromise UA dimensions in those who already have a predisposition to present small dimensions in these regions. Evaluating the patient as a whole, focusing on the functional improvement, is a responsibility of the orthodontist; therefore, decreasing chances of presenting future disorders should also be an objective of the treatment. Further longitudinal studies in different age groups are necessary to adequately characterize UA during growth and development, and aging. 

## CONCLUSION

MRP did not present significant differences for the tV, AxMin and morphology between different anteroposterior craniofacial skeletal patterns. When VP was assessed separately, there were differences between Class II and Class III patients for the tL and tV measures. Class II subjects have smaller tV in the VP region. In general, tV and AxMin tended to decrease in all evaluated regions when ANB angle values increased.
